# Malting Period Effect on the Phenolic Composition and Antioxidant Activity of Finger Millet (*Eleusine coracana* L. Gaertn) Flour

**DOI:** 10.3390/molecules23092091

**Published:** 2018-08-21

**Authors:** Henry O. Udeh, Kwaku G. Duodu, Afam I. O. Jideani

**Affiliations:** 1 Department of Food Science and Technology, School of Agriculture, University of Venda, Private Bag X5050, Thohoyandou 0950, South Africa; okwudilichukwudeh@yahoo.com; 2 Department of Food Science, Faculty of Natural and Agricultural Sciences, University of Pretoria, Private Bag X20, Hatfield 0028, South Africa; gyebi.duodu@up.ac.za

**Keywords:** *Eleusine coracana*, malting, phenolic compounds, antioxidant activity, UPLC-MS

## Abstract

The present study examined the influence of malting on the phenolic composition of two cultivars of finger millet using an ultra-performance liquid chromatography mass spectrometer. Total polyphenols and antioxidant activities of the grains were also evaluated using sorghum as an external reference. Catechin, epicatechin, quercetin, taxifolin, and hesperitin were isolated flavonoids, whereas protocatechuic acid was the phenolic acid detected in finger millet malt. Increases in the content of catechin, epicatechin, and protocatechuic acid were observed for 72 h and 96 h for brown finger millet and sorghum malt. Complete loss of taxifolin and hesperitin were observed with the malting period for finger millet cultivars. A similar loss was noted in the proanthocyanidin A1/A2 and catechin content of sorghum with malting time. The grain malt exhibited 2,2-diphenyl-1-picrylhydrazyl,2,2′-azinobis-3-ethylbenzthiazoline-6-sulfonic acid (ABTS) radical scavenging and iron reducing activities. Increased ABTS and iron reducing activity with malting time were observed for the finger millet cultivars. The study demonstrates the presence of hesperitin in finger millet, and also shows that 72 h and 96 h of malting enhanced the catechin, epicatechin, and protocatechuic acid content, in addition to the antioxidant activity of the grain.

## 1. Introduction

Finger millet, *Eleusine coracana* (L.) Gaertn, has been perceived as a potential “super grain” by the United States National Academies as one of the most nutritious among all the major cereal grains [1,2,3,4]. It is a small seeded subsistence food crop belonging to the grass family *Poaceae*. The nutrient-rich grain is mainly used for making unleavened bread, among other preparations like cakes and puddings, as well as stiff and thin porridges. Besides its use for brewing, the malted flour is also used in the preparation of infant and geriatric foods, and as a popular food supplement for diabetics [1,2]. Finger millet is gluten-free, which is ideal for patients suffering from celiac disease [5,6]. Regular consumption of finger millet has been linked with a reduced risk of diabetes mellitus, hypercholesterolemia, prevention of the oxidation of low-density lipoproteins, and in the improvement of gastrointestinal health [4,7,8]. These unique health beneficial properties have been attributed to the phenolic compounds present in the grain, as well as its dietary fibre content. The plant itself has been found to have diaphoretic, diuretic, and anthelmintic properties, and the leaf juice has been used for health-improvement for women at childbirth [9]. It is also used as a folk remedy for treatments including leprosy, liver disease, measles, pleurisy, pneumonia, and small pox [9]. Finger millet, however, is underutilised due to its minimal inclusion in convenience food products, unawareness of the public, lack of research, and novel product development processes [1,3,10].

Phenolic compounds are of great importance for food and beverages derived from plants, since these compounds are responsible for these products’ nutraceutical and organoleptic properties. These compounds are, therefore, closely linked with the quality of such products, and thus their analysis is of significant interest. Sorghum (*Sorghum bicolor* L. Moench) is the fifth most utilised cereal in the world and is particularly important as a food source for populations in Africa and Asia [11,12]. Sorghum belongs to the grass family Poaceae, formally known as Gramineae. It is a rich source of various phytochemicals including tannins, phenolic acids, anthocyanins, phytosterols, and policosanols [13]. Sorghum malt is widely used in the preparation of a variety of food products that are documented for their health properties [14,15,16]. Similar information on finger millet, however, is limited or not available, particularly in Southern Africa. Some available studies on malted finger millet have indicated the presence of phenolic acids, mainly the hydroxybenzoic acids along with hydroxycinnamic acids. Chethan et al. [17] investigated the effect of finger millet malt polyphenols on starch hydrolysis. In the study, phenolic acids were the major compounds reported, along with a flavonoid-quercetin. In other studies performed on finger millet malt, only phenolic acids were identified; however, data on the flavonoids of malted finger millet are very limited and inconclusive [18,19,20]. Flavonoids and their metabolites are important dietary phenolic compounds which are increasingly being investigated in many epidemiological studies for their possible role in the protection against chronic diseases [21]. In plants, flavonoids are involved in protection against UV radiation, oxidation and temperature stress, early plant development, signaling, and protection from insect and mammalian infestation [22]. Here, we report on the application of an ultra-performance liquid chromatography mass spectrometer as a tool to describe the phenolic composition of two local cultivars of finger millet, namely, brown and dark brown finger millet. Changes in the total polyphenols and antioxidant activities of the grain malts were also monitored.

## 2. Results

### 2.1. Composition and Changes in Phenolic Compounds of Finger Millet and Sorghum Malt

Table 1 shows the retention time and mass spectra characteristics of phenolic compounds in finger millet and sorghum malt using formic acid-methanol and HCl-methanol. The phenolic compounds identified in the extracts of finger millet and sorghum malt were flavan-3-ols (catechin, epicatechin), flavononol (taxifolin), flavonols (quercetin), proanthocyanidins (proanthocyanidin A1/A2), flavanones (hesperitin), and benzoic acid derivative (protocatechuic acid). Mass spectra and UPLC chromatograms of the phenolic compounds found in finger millet and sorghum grain malt extracts are shown in the appended Supplementary Materials.

Changes in the phenolic compounds of finger millet and sorghum during malting are shown in Table 2. Catechin was the predominant flavonoid found in the finger millet malt samples, followed by epicatechin and taxifolin for the formic acid-methanol extract. Taxifolin was not detected in the dark brown finger millet (DBFM) extract which, however, was found in the brown finger millet (BFM) extract. Malting for 24 h decreased the taxifolin content of the BFM extract to the point of non-detection; taxifolin, however, was detected in commensurable amounts in sorghum. Unlike the BFM extract, an increase in the taxifolin content of the sorghum extract was noted at 24 h of malting, but beyond this period, there was no significant change. Protocatechuic acid was the only phenolic acid detected in the malt samples. A decrease in total individual phenolic compounds for the DBFM extract was observed for up to 96 h of malting, particularly for protocatechuic acid (7.27 to 4.38 µg/g) and epicatechin (16.18 to 11.34 µg/g) (Table 2). Decreases were also observed in the total individual phenolic compounds of BFM and sorghum malt extracts at 24 and 48 h of malting; these later showed increases at 72 and 96 h of malting for protocatechuic acid, catechin, and epicatechin.

Hesperitin was found in a high amount, followed by quercetin for HCl-methanol extracts of the grain samples. A high amount of hesperitin (1636.9 µg/g) was recorded for the unmalted sorghum compared to BFM (96.7 µg/g) and DBFM (96.3 µg/g), respectively (Table 2). A decrease in total individual phenolic compounds was observed after 96 h of malting BFM and sorghum. There was a decrease in the total individual phenolic compound of DBFM for up to 48 h of malting, beyond which there was no significant change. Loss of catechin for the finger millet cultivars was observed at 24 h of malting and at 48 h for sorghum; a similar loss was observed in hesperitin for the 24 h BFM malt extract. A decrease and loss of hesperitin was observed at 24 h and 48 h for the DBFM malt extract, and a decrease in quercetin for up to 48 h of malting was observed for DBFM, beyond which there was no significant change. There was no statistical change in the quercetin content of BFM during the malting period, while a decrease in quercetin content was observed for up to 96 h of malting for sorghum.

### 2.2. Total Phenolic and Flavonoid Content of the Finger Millet and Sorghum Grain Varieties

The total phenolic content (TPC) of the unmalted and malted finger millet and sorghum samples is presented in Table 3. The TPC of DBFM, BFM, and sorghum samples ranged from 3.90–4.68, 4.41–7.09, and 17.23–19.26 mg GAE/g, respectively. The sorghum had higher TPC compared to the finger millet cultivars. A statistically insignificant (*p* > 0.05) increase in the TPC of sorghum was noted at 24 h of malting, which was not significantly different for other malting periods. A similar observation was noted for the DBFM extract; however, for the BFM extract, a significant decrease in TPC was observed at 24 and 48 h of malting, which later increased and remained constant, up to 96 h of malting.

The total flavonoid content (TFC) of the finger millet and sorghum samples is presented in Table 3, and it ranged from 0.35–0.95 mg QE/g in DBFM, 1.25–5.45 mg QE/g in BFM, and 22.15–28.37 mg QE/g in sorghum. Malting did not result in any significant change (*p* > 0.05) in the TFC of sorghum; however, a four-fold decrease in the TFC of BFM malt was observed at 24 h up to 72 h, which later increased at 96 h of malting. A slight increase in TFC was observed at 24 h for DBFM malt, which later decreased and remained constant for up to 96 h of malting. Generally, the TPC and TFC were found to concentrate at 24 h malt of the samples.

### 2.3. DPPH (2,2-Diphenyl-1-picrylhydrazyl) Free Radical Scavenging Activity of the Finger Millet and Sorghum Grain Varieties

The unmalted and malted grain samples showed DPPH radical scavenging activity which ranged from 46.79–70.16% in DBFM, 48.05–60.82% in BFM, and 54.73–65.89% in sorghum (Table 4). Malting did not result in a significant change (*p* > 0.05) in DPPH radical scavenging activity of BFM and sorghum; however, increased DPPH radical scavenging activity was observed for the 24 h DBFM malt extract.

### 2.4. ABTS (2′-Azinobis-3-ethylbenzthiazoline-6-sulfonic acid) Radical Scavenging Activity of the Finger Millet and Sorghum Grain Varieties

The antioxidant capacity of finger millet and sorghum malt determined by the ABTS radical cation ranged from 9.78–10.32 µM TE/g in DBFM, 9.76–10.63 µM TE/g in BFM, and 9.79–10.26 µM TE/g in sorghum (Table 4). Malting for 24 h resulted in a significant (*p* < 0.05) decrease in the ABTS radical quenching activity of DBFM and sorghum malt, which remained constant up to 96 h for DBFM malt. Increased ABTS radical quenching activity was noted at 48 h in sorghum malt, which remained constant up to 96 h of malting. No significant change in ABTS radical quenching activity was observed for 24 h BFM malt; however, an increase was noted at 48 h BFM malt, which remained constant up to 96 h.

### 2.5. Ferric Reducing Antioxidant Activity of the Finger Millet and Sorghum Grain Varieties

The finger millet and sorghum malt showed iron reducing activity which ranged from 0.7975–0.9798 in DBFM, 0.9199–0.9961 in BFM, and 1.0101–1.0375 in sorghum (Table 4). Sorghum showed a higher iron reducing activity compared to the finger millet cultivars, both for the unmalted and malted grains. Increased iron reducing activity with malting time was observed for up to 96 h BFM malt. A decrease in iron reducing activity was observed within 24 and 48 h of DBFM malt, which later increased significantly at 96 h of malting.

## 3. Discussion

Literature on the extraction method used for finger millet polyphenols so far, has shown that most of the studies used 1% HCl in methanol for extraction of the polyphenols with different periods and modes of extraction [17,18,19,20,23,24,25,26,27,28]. In a study of Chethan and Malleshi [28], it was found that the extractability of finger millet polyphenols with different polar solvents varied considerably and acidifying the organic solvents improved the levels of the extraction. It was noted that 1% HCl-methanol was the most suitable among the different solvents tested for the extraction of the polyphenols. To the best of our knowledge, no other acidifying agent has been used in the extraction of finger millet polyphenols. Formic acid was tested based on its wide application in the profiling of fruit and wine polyphenols, which are predominantly flavonoids. The results show that the extraction of finger millet and sorghum malt flour with formic acid affected the phenolic composition of the grains.

Studies on malted finger millet phenolics have demonstrated the presence of a variety of phenolic acids for aqueous solvents acidified with 1% HCl using HPLC [17,19,20]. The results of this study revealed the presence of more flavonoids than phenolic acids in finger millet malt than previously reported. Surprisingly, hesperitin, which is an important dietary flavonoid present in fruits, was found in the finger millet extract obtained using 1% HCl-methanol. The finger millet extract obtained using formic acid-methanol, however, had more flavonoids compared to the extracts obtained with 1% HCl-methanol. Ivanova et al. [29] observed that the use of a lower concentration of HCl for the extraction of phenolic compounds has a protective effect on flavonoids. It was observed that 0.1% HCl does not cause degradation as much as 1% HCl, which induces hydrolysis or the degradation of flavonoids, hence it is suggested that formic acid facilitates the isolation of flavonoids of finger millet better than hydrochloric acid. In this regard, further studies are required to investigate the effect of different acidification and instruments of extraction in order to further describe the phenolic composition of finger millet.

A decrease in the phenolic compounds for the finger millet and sorghum samples was observed, particularly at 24 and 48 h of malting. A decrease and complete loss of hesperitin and taxifolin at and after 24 h of malting was noted. The changes can be attributed to degradation or bioconversion of the phenolic compound to other active or less active forms during malting, as well as the loss of hydrolysed phenolic compounds through leaching during steeping and germination. The findings agree with other studies where phenolic compounds were observed to be either destroyed or converted to other forms during the germination of cereal grains [19,20]. Conversely, increases were noted at 72 and 96 h of malting, which varied for the finger millet and sorghum cultivars. A similar observation was reported for finger millet malt [17,19,20]. The observed increase in phenolic compounds of the malt samples could be due to the increased activity of induced endogenous enzymes on various bound or conjugated phenolic compounds and/or the bioconversion of phenolic compounds to other forms. Furthermore, varietal differences could also have affected the changes in the phenolic compounds of finger millet and sorghum malt.

The total phenolic and flavonoid content of the finger millet is comparable to the report of Hithamani and Srinivasan [19], who found 10.2 and 5.54 mg/g for total phenolic and total flavonoid content in finger millet, respectively. The total phenolic and flavonoid content of the finger millet cultivars was found to concentrate in the 24 h malt compared to the other malting periods. Changes in total polyphenols of cereal grains are known to result from a variety of enzymatic hydrolysis, bioconversion, and oxidative degradation during processing, such as soaking, germination, roasting, and fermentation. The decrease in total phenolic and total flavonoid contents noted within 48 h to 96 h of malting of the finger millet cultivars could be attributed to the loss of hydrolysed phenolic compounds during the germination periods.

The antioxidant activity of finger millet has been studied by several researchers; however, information on the antioxidant activity of malted finger millet is scarce [19,25]. The finger millet and sorghum malt exhibited DPPH, ABTS radical scavenging, and iron reducing activities. Malting did not result in any significant (*p* < 0.05) increase in DPPH radical scavenging activity for BFM and sorghum, except for DBFM, which was significantly (*p* < 0.05) higher at 24 h of malting. Similar changes were noted in the report of Subba Rao and Muralikrishna [19]. In their study, high antioxidant activity was noted for bound phenolic acids of 24 h malted finger millet compared to other malting periods. A concentration-dependent DPPH radical scavenging activity was not observed, as expected, for the sorghum, despite its higher total polyphenol content compared to the finger millet cultivars. The malting process did not bring about any significant change in ABTS radical scavenging activity of the malt samples beyond 48 h of malting. An increased iron reducing activity was observed for the finger millet cultivars, which was significantly (*p* < 0.05) higher at 96 h of malting. A similar finding was reported by Subba Rao and Muralikrishna [19], where high antioxidant activity for free phenolic acids was recorded for 96 h finger millet malt. In a whole grain cereal, there are different substances that contribute directly or indirectly to the antioxidant activities. Polyphenol content of plant foods is well known to have a parallel effect on the antioxidant activity of plant foods, whereas vitamin E and minerals, such as iron, zinc, manganese, selenium, and copper, act as a co-factor in antioxidant enzymes; and sulphur-containing amino acids, methionine and cysteine, are precursors of glutathione, an endogenous antioxidant [11,30]. The changes noted in the antioxidant activities of the finger millet and sorghum malt, could thus be attributed to the total phenolic content, released minerals, and other organic compounds with an antioxidant effect. The observed changes in the iron reducing activity of the finger millet cultivars could not be entirely attributed to the total polyphenol content, but also to other components of the malt with antioxidant properties. The finding supports the fact that a high total phenolic content does not necessarily translate into high antioxidant activity, but is a result of a synergy between total phenols, released organic components, and minerals with antioxidant properties.

## 4. Materials and Methods

### 4.1. Cereal Grain Samples

Local cultivars of finger millet (FM), brown (BFM) and dark brown (DBFM), as well as red-coloured sorghum, were purchased from retail outlets in Thohoyandou, Limpopo Province, South Africa. The reagents Folin-Ciocalteu’s, gallic acid and quercetin standards, 2,2-diphenyl-1-picrylhydrazyl (DPPH), and 2′-azinobis-3-ethylbenzthiazoline-6-sulfonic acid (ABTS) were purchased from Rochelle Chemicals, Johannesburg, South Africa. Solvents and phenolic standards used were of analytical ultra-performance liquid chromatography mass spectrometer (UPLC-MS) grade.

### 4.2. Malting of the Cereal Grain Varieties

The finger millet and sorghum cultivars were malted according to the method of Chethan et al. [17]. Two (2) varieties of finger millet, namely brown finger millet (BFM) and dark brown finger millet (DBFM), were used in this study, while sorghum was used an as external reference. Sorghum was used as an external reference in the present study in an attempt to prepare a comparable finger millet malt flour that could be used in malted and fermented foods and beverages, particularly in Southern Africa. The food grains were cleaned before use. One hundred gram (100 g) portions of the finger millet and sorghum samples were initially soaked in water for 24 h at 25 °C in a growth chamber; the grains were spread on a clean cheese cloth and kept moist by sprinkling water periodically at intervals of 24 h. The sprouted grains were kilned for 8 h at 50 °C, after which a characteristic malt aroma was obtained. The kilned grains were milled to fine flour and stored in polyethylene bags at −20 °C until analysis.

### 4.3. Preparation of Polyphenol Extracts

One gram (1 g) of the cereal samples was refluxed in an ultrasonic bath with 15 mL of methanol containing 15% formic acid for 1 h at 40 °C. The extracts were centrifuged at 13,000 rpm for 6 min and then pipetted into vials and stored at 4 °C before analysis. Extraction was also done using 20 mL of methanol containing 1% HCl for 2 h at 60 ± 5 °C in a water bath. The mixtures were centrifuged at 5000 rpm for 20 min and the supernatants were separated and analysed for individual phenolic compounds. Finger millet and sorghum extracts obtained using 1% HCl-methanol were used for evaluating the total polyphenol content and antioxidant activities.

### 4.4. Identification and Quantification of Phenolic Compounds of the Cereal Grain Varieties

Separation and identification of the phenolic compounds in the extracts were carried out using a Waters Synapt G2 quadrupole time-of-flight mass spectrometer (MS). It was fitted with a Waters Ultra performance liquid chromatograph (UPLC) and photo diode array detection. Separation was achieved on a Waters BEH C18, 2.1 × 100 mm column with 1.7 µm particles. A gradient was applied using 0.1% formic acid (solvent A) and acetonitrile containing 0.1% formic acid (solvent B). The gradient started at 100% solvent A for 1 min and changed to 28% B over 22 min in a linear way. It then went to 40% B over 50 s and a wash step of 1.5 min at 100% B, followed by re-equilibration to initial conditions for 4 min. The flow rate was 0.3 mL/min and the column was kept at 55 °C. The injection volume was 2 µL. Data was acquired in MS^E^ mode, which consisted of a low collision energy scan (6 V) from *m*/*z* 150 to 1500 and a high collision energy scan, from *m*/*z* 40 to 1500. The high collision energy scan was done using a collision energy ramp of 30–60 V. The photo diode array detector was set to scan from 220–600 nm. The mass spectrometer was optimized for the best sensitivity, with a cone voltage of 15 V, the desolvation gas was nitrogen at 650 L/h, and a desolvation temperature of 275 °C was employed. The instrument was operated with an electrospray ionization probe in the negative mode. Sodium formate was used for calibration and leucine encephalin was infused in the background as lock mass for accurate mass determination. Individual peaks and phenolic compounds were identified by comparing the retention time and spectra of each peak with known standards under identical conditions.

### 4.5. Total Phenolic Content of the Cereal Grain Varieties

Total phenolic content of the sample extracts were determined according to the method of Singleton et al. [31]. Briefly, 0.1 mL of the acidified methanolic extract was mixed with 5 mL distilled water in a 50 mL volumetric flask. Folin-Ciocalteu’s reagent (1:2 dilution with water) (2.5 mL) and 7.5 mL 15% sodium carbonate solution was added, mixed thoroughly, made up to 50 mL, and allowed to react for 30 min. The absorbance of the reaction mixture was read at 760 nm with a 96 well microplate spectrophotometer. A calibration curve was prepared using a standard solution of gallic acid and the result was expressed as mg of gallic acid equivalent (GAE), per g of the sample.

### 4.6. Total Flavonoid Content of the Cereal Grain Varieties

Total flavonoid content was determined spectrophotometrically according to Zhishen et al. [32]. The extract (0.1 mL) was mixed with 4.9 mL distilled water and 0.3 mL 5% (*w*/*v*) NaNO_2_ was added. After 5 min, 0.3 mL 10% (*w*/*v*) AlCl_3_ and at 6 min, 2 mL 1 M NaOH, were added, and immediately, the volume was made up to 10 mL with distilled water. The mixture was vortexed and the absorbance was read at 510 nm. A calibration curve was prepared using quercetin as the standard. The result was expressed as mg quercetin equivalent (QE) per g of the sample.

### 4.7. DPPH (2,2-Diphenyl-1-picrylhydrazyl) Free Radical Scavenging Activity of the Cereal Grain Varieties

The DPPH radical scavenging activity was determined according to the method of De Ancos et al. [33]. An aliquot (10 µL) of the acidified methanolic extract was mixed with distilled water (90 µL) and 3.9 mL of methanolic 0.1 mM DPPH solution. The mixture was thoroughly vortexed and kept in the dark for 30 min, and the absorbance was read at 515 nm. The result was expressed as percentage inhibition of the DPPH radical. The percentage of inhibition of the DPPH radical was calculated according to the following equation:

% Inhibition of DPPH = [Abs control − Abs sample/Abs control] × 100

where Abs control is the absorbance of the DPPH solution without the extract.

### 4.8. ABTS (2′-Azinobis-3-ethylbenzthiazoline-6-sulfonic acid) Radical Scavenging Activity of the Finger Millet and Sorghum Grain Varieties

The ABTS radical scavenging activity of the unmalted and malted samples was determined according to the methods of Arnao et al. [34] and Thaipong et al. [35], with slight modification. Equal volumes of 7.4 mM 2,2′-azinobis-3-ethylbenzthiazoline-6-sulfonic acid (ABTS) and 2.6 mM potassium persulfate, both prepared in distilled water, were mixed and allowed to react for 12 h, at room temperature, in the dark, to obtain an ABTS radical cation. The solution was then diluted by mixing 1 mL ABTS solution with 60 mL of pH 7.4 phosphate buffer containing 0.1 M NaOH to obtain an initial absorbance of 0.098 ± 0.00 at 734 nm. Fresh extracts (150 µL) of the cereal samples were allowed to react with 2850 µL of the ABTS^•+^ solution for 5 min and the absorbance was read at 734 nm. The standard curve was prepared by dissolving Trolox in phosphate buffer (pH 7.4), and reacting it with 2850 µL of the ABTS^•+^ solution for 5 min. The results were expressed as µM Trolox equivalent (TE)/g.

### 4.9. Ferric Reducing Antioxidant Power of the Cereal Grain Varieties

The reducing power assay was determined according to the method of Oyaizu [36]. Briefly, 100 µL of the extract was placed in a test tube and the volume was adjusted to 1 mL with methanol. Phosphate buffer (2.5 mL 0.2 M, pH 6.6) and 2.5 mL 1% potassium ferricyanide were added to the tube and vortexed. The mixture was left for 20 min at 50 °C, in a water bath. After incubation, 2.5 mL 10% (*w*/*v*) trichloroacetic acid was added and the mixture was centrifuged at 5000 rpm for 20 min. Then, 2.5 mL of the supernatant was taken and mixed with 2.5 mL distilled water and 0.5 mL 0.1% (*w*/*v*) ferric chloride in a test tube, and the absorbance was measured at 700 nm. A higher absorbance value indicates a higher reducing power.

### 4.10. Statistical Analysis

Data obtained were subjected to a one way ANOVA by Duncan’s multiple comparison test using SPSS version 24.0 (SPSS Inc., Chicago, IL, USA). The mean values were considered to be statistically significant at *p* < 0.05.

## 5. Conclusions

UPLC-MS data for the acidification methods used for extraction of the finger millet polyphenols show more flavonoids in finger millet malt than was previously reported. The presence of hesperitin in finger millet was established in the study. Decrease and loss of hesperitin and taxifolin were observed with malting time, whereas catechin, epicatechin, quercetin, and protocatechuic acid showed increases at 72 and 96 h of malting, which is beyond the 48 h of malting commonly used in finger millet malt preparation. Total polyphenol content was found to concentrate in the 24 h malt samples. The finger millet malt exhibited antioxidant activities, which increased with malting period for the iron reducing activity. Varietal differences were found to play important roles in how malting affects the phenolic compounds of the cereal grains. The data presented further substantiate the health associated claims of finger millet consumption with regards to its flavonoid components, and also on malting conditions that could be useful in enhancing important phenolic compounds and antioxidant activity of the grain for food, beverage, and therapeutic preparations.

## Figures and Tables

**Table 1:** Retention time and MS characteristics of phenolic compounds identified in finger millet and sorghum.

Extraction Solvents	*t*_R_ (min)	[M − H]^−^ (*m*/*z*)	MS/MS Fragments (Intensity %)	Identified Compounds
15% Formic acid—methanol	*Benzoic acid derivatives*			
	7.87	153	153 (100)	Protocatechuic acid
	*Flavan-3-ols*			
	11.17	289	289 (100), 245 (30), 203 (21), 123 (19), 109 (14)	Catechin
	13.32	289	289 (100), 245 (40), 203 (31), 137 (40)	Epicatechin
	*Flavononol*			
	17.05	303	303 (100), 285 (11), 236 (9), 191 (4), 175 (5)	Taxifolin
1% HCl-methanol	*Flavan-3-ols*			
	12.22	289	289 (100), 245 (84), 209 (72), 177(61), 137(47), 109 (37)	Catechin
	*Proanthocyanidins*			
	10.20	575	575 (100)	Proanthocyanidin A1/A2
	*Flavonols*			
	24.18	303	303 (100)	Quercetin
		*Flavanones*			
		21.88	301	301 (100), 269 (89)	Hesperitin

*t*_R_ = retention time; [M − H]^−^ = negative ionic mode; MS = mass spectra.

**Table 2:** Changes in contents (µg/g) of phenolic compounds in formic acid and HCl-methanol extracts of the cereal grain samples.

Grains/Compounds	Malting Period (h)										
	15% Formic Acid-Methanol						1% HCl-Methanol				
	0	24	48	72	96		0	24	48	72	96
*Dark brown finger millet*											
PA	7.3 ± 0.1 ^a^	6.7 ± 0.2 ^b^	5.9 ± 0.0 ^c^	4.9 ± 0.2 ^d^	4.4 ± 0.1 ^e^		Nd	Nd	Nd	Nd	Nd
CA	25.9 ± 0.4 ^a^	24.8 ± 0.6 ^a^	25.0 ± 0.5 ^a^	23.2 ± 0.6 ^b^	22.1 ± 0.0 ^b^		10.4 ± 0.6 ^a^	Nd	Nd	Nd	Nd
ECA	16.2 ± 0.1 ^a^	14.7 ± 0.3 ^b^	13.8 ± 0.3 ^b^	11.5 ± 0.9 ^c^	11.3 ± 0.2 ^c^		Nd	Nd	Nd	Nd	Nd
QE	Nd	Nd	Nd	Nd	Nd		46.7 ± 2.9 ^a^	41.7 ± 1.3 ^b^	23.3 ± 1.6 ^c^	25.1 ± 2.8 ^c^	27.8 ± 1.9 ^c^
Hp	Nd	Nd	Nd	Nd	Nd		96.3 ± 13.8 ^a^	9.2 ± 3.7 ^b^	Nd	Nd	Nd
PAC A1	Nd	Nd	Nd	Nd	Nd		Nd	Nd	Nd	Nd	Nd
PAC A2	Nd	Nd	Nd	Nd	Nd		Nd	Nd	Nd	Nd	Nd
Total	49.4 ± 0.6	46.2 ± 1.1	44.7 ± 0.8	39.6 ± 1.7	37.8 ± 0.3		153.4 ± 17.3	50.9 ± 5.0	23.3 ± 1.6	25.1 ± 2.8	27.8 ± 1.9
*Brown finger millet*											
PA	14.8 ± 0.0 ^a^	4.3 ± 0.1 ^b^	2.8 ± 0.0 ^c^	4.7 ± 0.1 ^d^	4.6 ± 0.2 ^d^		Nd	Nd	Nd	Nd	Nd
CA	27.9 ± 0.3 ^a^	20.7 ± 0.4 ^b^	17.9 ± 0.4 ^c^	20.9 ± 0.1 ^b^	21.9 ± 0.0 ^d^		1.4 ± 0.0 ^a^	Nd	Nd	Nd	Nd
ECA	17.4 ± 0.8 ^a^	9.8 ± 0.4 ^b^	7.7 ± 0.1 ^c^	10.4 ± 0.2 ^b^	10.4 ± 0.1 ^b^		Nd	Nd	Nd	Nd	Nd
QE	Nd	Nd	Nd	Nd	Nd		66.2 ± 4.4 ^a^	54.2 ± 1.8 ^a^	43.6 ± 4.9 ^a^	42.1 ± 2.4 ^a^	38.9 ± 5.7 ^a^
TX	0.4 ± 0.3 ^a^	0.11 ± 0.03 ^a^	Nd	Nd	Nd		Nd	Nd	Nd	Nd	Nd
Hp	Nd	Nd	Nd	Nd	Nd		96.7 ± 35.7 ^a^	Nd	Nd	Nd	Nd
PAC A1	Nd	Nd	Nd	Nd	Nd		Nd	Nd	Nd	Nd	Nd
PAC A2	Nd	Nd	Nd	Nd	Nd		Nd	Nd	Nd	Nd	Nd
Total	60.5 ± 1.4	34.9 ± 0.9	28.4 ± 0.5	36.00 ± 0.40	36.9 ± 0.3		164.3 ± 40.1	54.2 ± 1.8	43.6 ± 4.9	42.1 ± 2.4 ^a^	38.9 ± 5.7 ^a^
*Sorghum*											
PA	5.0 ± 0.5 ^a^	2.1 ± 0.0 ^b^	1.9 ± 0.0 ^b^	3.2 ± 0.03 ^c^	3.2 ± 0.1 ^c^		Nd	Nd	Nd	Nd	Nd
CA	0.6 ± 0.0 ^a^	0.3 ± 0.0 ^b^	0.3 ± 0.0 ^b^	0.3 ± 0.01 ^b,c^	0.4 ± 0.0 ^c^		24.6 ± 5.8 ^a^	9.2 ± 2.3 ^b^	Nd	Nd	Nd
ECA	2.9 ± 0.2 ^a^	3.9 ± 0.0 ^b,d^	3.8 ± 0.0 ^b^	4.4 ± 0.08 ^c^	4.3 ± 0.0 ^c,d^		Nd	Nd	Nd	Nd	Nd
QE	Nd	Nd	Nd	Nd	Nd		84.9 ± 5.6 ^a^	63.4 ± 1.7 ^b^	41.9 ± 3.5 ^c^	37.0 ± 1.9 ^c^	23.1 ± 1.9 ^d^
TX	3.4 ± 0.9 ^a^	4.5 ± 1.2 ^b,c^	4.1 ± 1.0 ^b^	4.7 ± 0.7 ^b,c^	4.6 ± 0.7 ^b,c^		Nd	Nd	Nd	Nd	Nd
Hp	Nd	Nd	Nd	Nd	Nd		1636.9 ± 241.4 ^a^	1210.0 ± 256.0 ^b^	244.5 ± 87.8 ^c^	52.50 ± 15.2 ^d^	32.9± 8.4 ^e^
PAC A1	Nd	Nd	Nd	Nd	Nd		22.1 ± 3.3 ^a^	13.5 ± 8.3 ^a^	Nd	Nd	Nd
PAC A2	Nd	Nd	Nd	Nd	Nd		26.9 ± 4.5 ^a^	20.5 ± 6.8 ^a^	4.2 ± 0.0 ^b^	Nd	Nd
Total	8.5 ± 0.7	6.3 ± 0.0	6.0 ± 0.0	7.9 ± 0.12	7.9 ± 0.1		1795.4 ± 260.6	1316.6 ± 275.1	290.6 ± 91.3	89.5 ± 17.1	56.0 ± 10.3

PA, protocatechuic acid; CA, (+)-catechin; ECA, (−)-epicatechin; QE, quercetin; TX, taxifolin; Hp, hesperitin; PAC A1, proanthocyanidin A1; PAC A2, proanthocyanidin A2; nd, not detected; values given are the mean of triplicate experiments; mean values in a row with different superscripts are significantly different at *p* < 0.05.

**Table 3:** Effect of malting period on total phenolic and total flavonoid content of finger millet and sorghum extracts obtained using 1% HCl-methanol.

**Total Phenolic Content (mg GAE/g)**					
**Grain Samples**	**0**	**24 h**	**48 h**	**72 h**	**96 h**
Dark brown finger millet	4.42 ± 0.30 ^a,z^	4.68 ± 0.00 ^a,y^	4.64 ± 0.14 ^a,y^	3.90 ± 0.41 ^a,z^	4.33 ± 0.22 ^a,y^
Brown finger millet	7.09 ± 0.49 ^a,y^	5.39 ± 0.05 ^b,y^	4.41 ± 0.16 ^c,y^	5.22 ± 0.36 ^b,c,y^	5.02 ± 0.14 ^b,c,y^
Sorghum	17.23 ± 1.09 ^a,x^	18.92 ± 2.27 ^a,x^	18.56 ± 0.47 ^a,x^	17.54 ± 0.38 ^a,x^	19.26 ± 0.13 ^a,x^
**Total Flavonoid Content (mg QE/g)**					
Dark brown finger millet	0.92 ± 0.15 ^a,y^	0.95 ± 0.07 ^a,y^	0.66 ± 0.07 ^a,b,y^	0.72 ± 0.07 ^a,b,y^	0.35 ± 0.19 ^b,y^
Brown finger millet	5.45 ± 0.27 ^a,y^	2.98 ± 0.33 ^b,y^	2.01 ± 0.15 ^c,y^	1.25 ± 0.17 ^d,y^	2.03 ± 0.18 ^c,y^
Sorghum	22.21 ± 3.49 ^a,x^	22.15 ± 3.37 ^a,x^	27.77 ± 1.56 ^a,x^	28.37 ± 2.99 ^a,x^	25.29 ± 2.28 ^a,x^

^a,b,c^ Mean within each row for each group not followed by the same superscript is significantly different (*p* < 0.05). ^x,y,z^ Mean within each column for each group not followed by the same superscript is significantly different (*p* < 0.05).

**Table 4:** Effect of malting period on antioxidant activity of finger millet and sorghum extracts obtained using 1% HCl-methanol.

**DPPH Radical Scavenging Activity (%)**					
**Grain Samples**	**0 h**	**24 h**	**48 h**	**72 h**	**96 h**
Dark brown finger millet	51.00 ± 3.34 ^a,x^	70.16 ± 2.03 ^b,z^	61.76 ± 3.48 ^a,c,x^	46.79 ± 9.71 ^c,x^	69.16 ± 5.98 ^a,c,x,z^
Brown finger millet	56.60 ± 1.69 ^a,x^	51.29 ± 1.97 ^a,y^	48.05 ± 7.64 ^a,x^	60.82 ± 7.64 ^a,x^	48.49 ± 1.46 ^a,x,y^
Sorghum	64.85 ± 8.31 ^a,x^	61.15 ± 2.39 ^a,x^	54.73 ± 1.53 ^a,x^	65.89 ± 5.13 ^a,x^	62.67 ± 6.51 ^a,x^
**ABTS (µM TE/g)**					
Dark brown finger millet	10.32 ± 0.07 ^a,y^	9.92 ± 0.05 ^b,x^	9.93 ± 0.04 ^b,y^	9.78 ± 0.21 ^b,y^	9.84 ± 0.08 ^b,y^
Brown finger millet	9.76 ± 0.10 ^a,x^	9.76 ± 0.15 ^a,x^	10.57 ± 0.02 ^b,x^	10.63 ± 0.11 ^b,x^	10.30 ± 0.05 ^b,x^
Sorghum	9.93 ± 0.11^a,b,x^	9.79 ± 0.15 ^b,x^	10.23 ± 0.03 ^a,x^	10.02 ± 0.19 ^ab,x^	10.26 ± 0.03 ^a,x^
**Iron Reducing Activity**					
Dark brown finger millet	0.89 ± 0.01 ^a,y^	0.85 ± 0.01 ^b,y^	0.79 ± 0.01 ^c,z^	0.96 ± 0.01 ^d,y^	0.98 ± 0.01 ^d,x^
Brown finger millet	0.99 ± 0.01 ^a,x^	0.92 ± 0.01 ^b,y^	0.94 ± 0.03 ^a,b,y^	0.95 ± 0.02 ^a,b,y^	0.99 ± 0.03 ^a,x^
Sorghum	1.04 ± 0.02 ^a,x^	1.02 ± 0.03 ^a,x^	1.01 ± 0.02 ^a,x^	1.02 ± 0.01 ^a,x^	1.01 ± 0.01 ^a,x^

^a,b,c^ Mean within each row for each group not followed by the same superscript is significantly different (*p* < 0.05). ^x,y,z^ Mean within each column for each group not followed by the same superscript is significantly different (*p* < 0.05).

## Supplementary Materials

Supplementary Materials are available online.
