# Supplementary material for: Malting Period Effect on the Phenolic Composition and Antioxidant Activity of Finger Millet (Eleusine coracana L. Gaertn) Flour
Source: Molecules. 2018 Aug 21;23(9):2091. doi: 10.3390/molecules23092091 (PMC6225115; doi:10.3390/molecules23092091)
Supplement: Supplementary file 1 [file molecules-23-02091-s001.pdf]

Fig. 1. MS/MS-fragments of identified phenolic compounds in the finger millet and sorghum malt. a) catechin, b) epicatechin, c) hesperitin, d) quercetin, e) proanthocyanidin A1/ A2, f) protocatechuic acid, g) taxifolin.

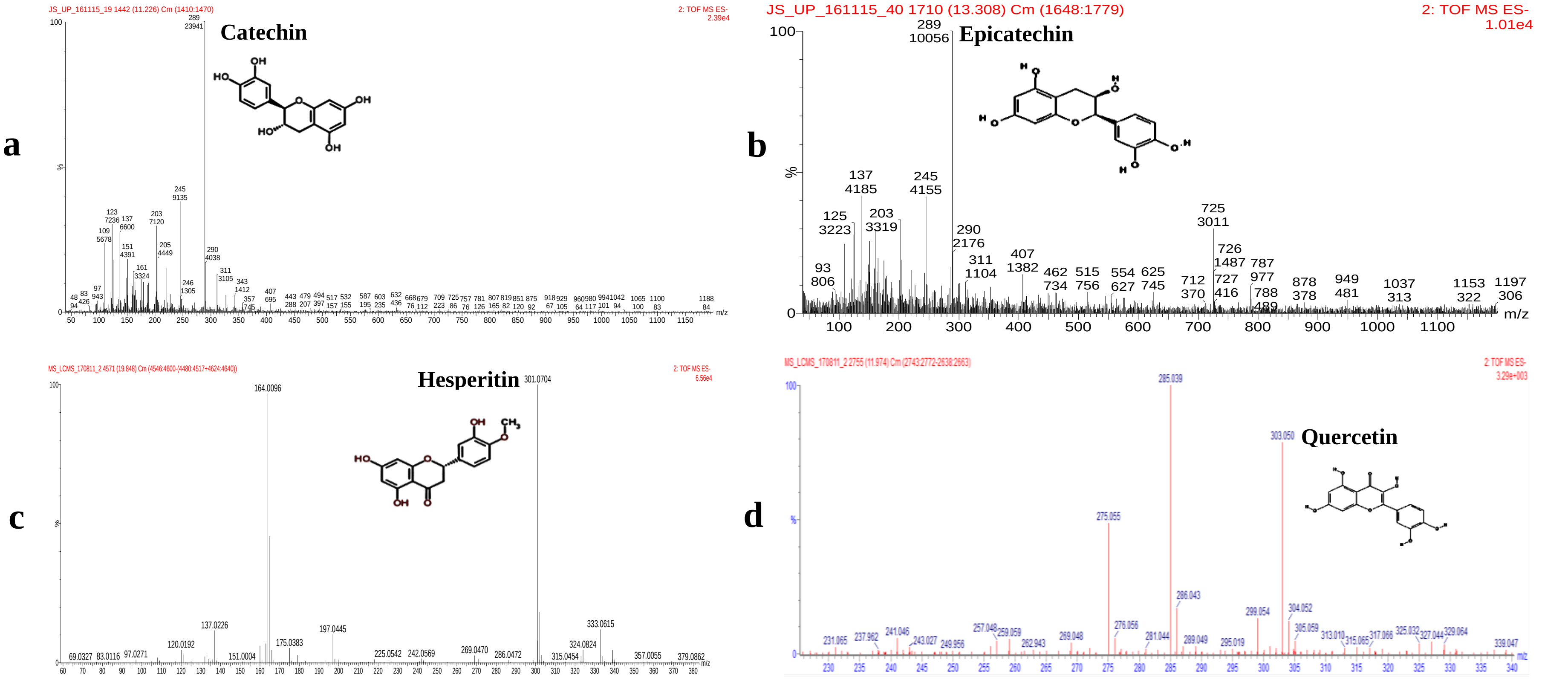

e

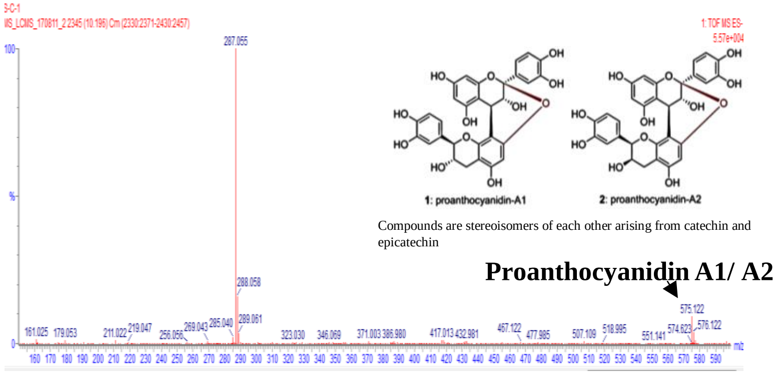
1: proanthocyanidin-A1
2: proanthocyanidin-A2
Compounds are stereoisomers of each other arising from catechin and epicatechin
Proanthocyanidin A1/ A2
1: TOF MS ES-
5.57e+004
m/z

f

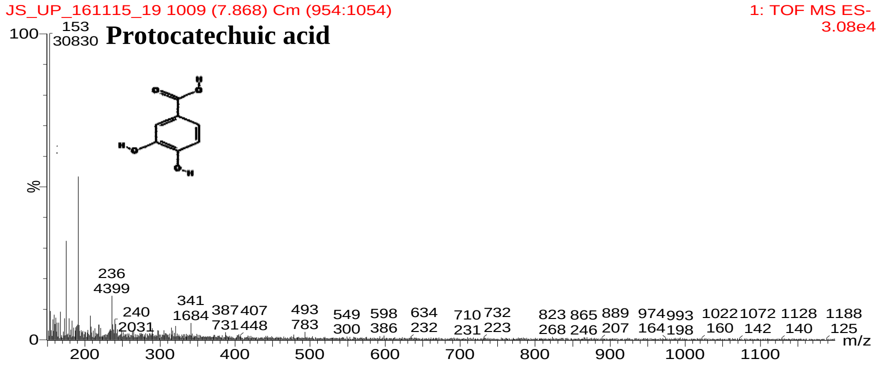
JS_UP_161115_19 1009 (7.868) Cm (954:1054)
1: TOF MS ES-
3.08e4
Protocatechuic acid
m/z

g

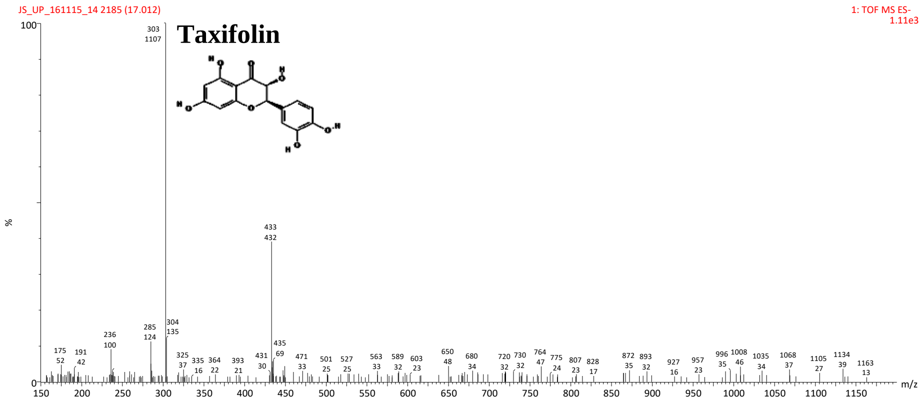
JS_UP_161115_14 2185 (17.012)
1: TOF MS ES-
1.11e3
Taxifolin
m/z

Fig. 2. Ultra-performance liquid chromatography mass spectrometer of the unmalted cereal grains. a) brown finger millet, b) dark brown finger millet, and c) sorghum.

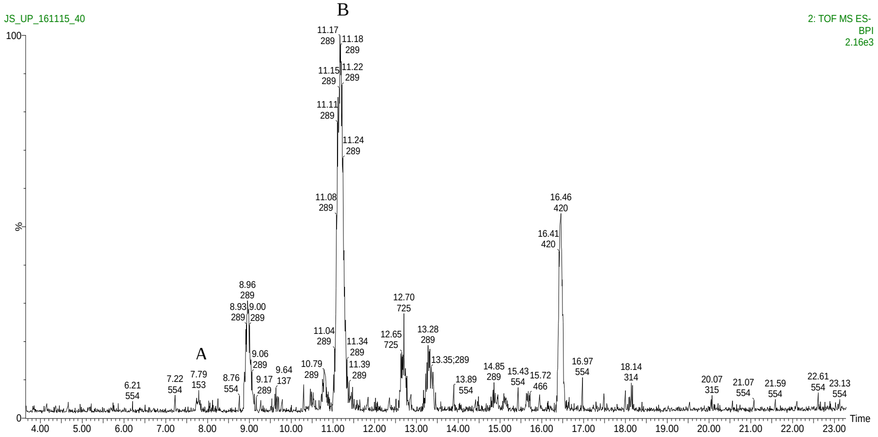

a. Brown finger millet

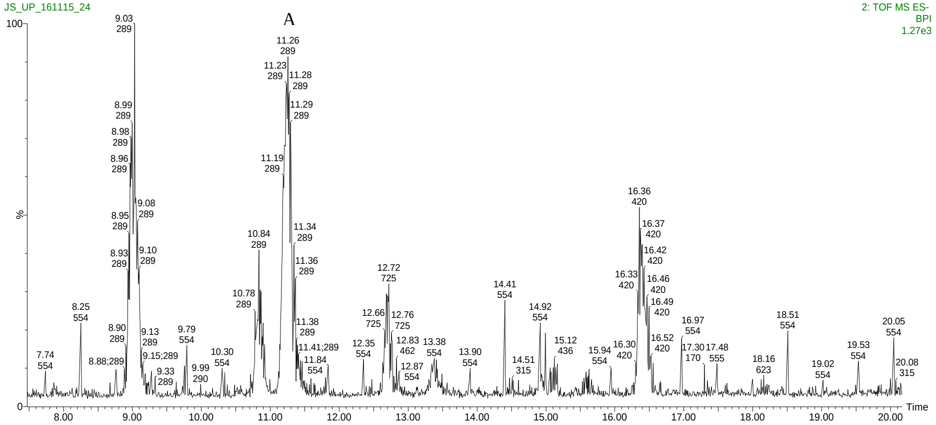

b. Dark brown finger millet

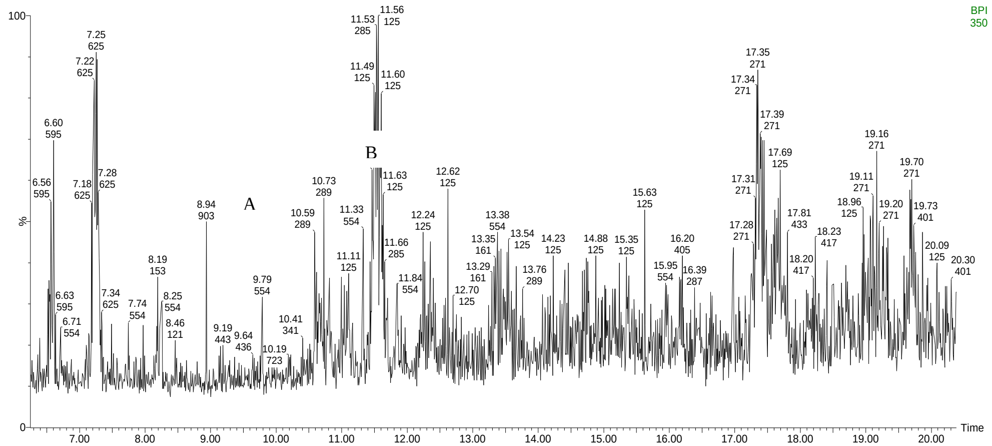

c. Sorghum
